# Direct-to-Patient Research: Piloting a New Approach to Understanding Drug Safety During Pregnancy

**DOI:** 10.2196/publichealth.4939

**Published:** 2015-12-22

**Authors:** Nancy A Dreyer, Stella CF Blackburn, Shahrul Mt-Isa, Jonathan L Richardson, Simon Thomas, Maja Laursen, Priscilla Zetstra-van der Woude, Anna Jamry-Dziurla, Valerie Hliva, Alison Bourke, Lolkje de Jong-van den Berg

**Affiliations:** ^1^ Quintiles Real-World & Late Phase Research Cambridge, MA United States; ^2^ Quintiles Real-World & Late Phase Research Reading United Kingdom; ^3^ Imperial College London United Kingdom; ^4^ Institute of Cellular Medicine Newcastle University Newcastle upon Tyne United Kingdom; ^5^ UK Teratology Information Service Newcastle Hospitals NHS Foundation Trust Newcastle upon Tyne United Kingdom; ^6^ Statens Serum Institut Copenhagen Denmark; ^7^ University of Groningen Groningen Netherlands; ^8^ Poznan University of Medical Sciences Poznan Poland; ^9^ Quintiles Real-World & Late-Phase Research St. Prex Switzerland; ^10^ IMS London United Kingdom

**Keywords:** pharmacovigilance, direct-to-patient, drug safety, validation

## Abstract

**Background:**

Little is known about the effects of human fetal exposure when a new drug is authorized unless it was specifically developed for use in pregnancy. Since many factors may contribute to adverse fetal effects, having comprehensive information about in utero exposures will enhance our ability to make correct determinations about causality.

**Objective:**

The objective of the study was to assess the extent to which women, recruited without the intervention of health care professionals (HCPs), will provide information, suitable for research purposes, via the Internet or by phone on some potential risk factors in pregnancy.

**Methods:**

To pilot direct-to-patient research for pharmacovigilance, we conducted a prospective, noninterventional study of medication use and lifestyle factors as part of the Pharmacoepidemiological Research on Outcomes of Therapeutics by a European ConsorTium (PROTECT) Consortium. Consenting women who self-identified as pregnant and residing in the United Kingdom (UK), Denmark (DK), The Netherlands, or Poland were recruited and could then choose to provide data every 2 or 4 weeks via the Internet or a telephonic interactive voice response system (IVRS). Self-reported drug use was compared with pharmacy register data in DK and with electronic health records in the UK.

**Results:**

Recruited women were on average older and more highly educated than the general population. Most respondents chose a frequency of every 4 weeks (56.99%, 1177/2065). Only 29.83% (464/1555) of women with due dates occurring during the study provided information on pregnancy outcome. For those responding by Internet, over 90.00% (1915/2065) reported using >1 pregnancy-related medication, 83.34% (1721/2065) reported using >1 other medicine, and 23.53% (486/2065) reported only over-the-counter medications, not counting herbals and dietary supplements. Some respondents (7.16%, 148/2065) reported that they chose not to take a prescribed medication (mostly medicines for pain or inflammation, and for depression) and 1.30% (27/2065) reported using medicines that had been prescribed to a friend or family member (oxycodone, paracetamol, and medications for acid-related problems). Relatively few respondents reported using fish oil (4.60%, 95/2065), other dietary supplements (1.88%, 39/2065), herbal products (7.07%, 146/2065), or homeopathic products (1.16%, 24/2065). Most medications for chronic conditions that were listed in the Danish prescription registry were also self-reported (83.3%, 145/174 agreement), with larger discrepancies for medications indicated for short-term use (54.0%, 153/283 agreement) and pregnancy-related medications (66.1%, 78/118).

**Conclusions:**

Self-reported information on medication use as well as other potential teratogenic factors can be collected via the Internet, although recruitment costs are not insubstantial and maintaining follow-up is challenging. Direct data collection from consumers adds detail, but clinical input may be needed to fully understand patients’ medical histories and capture birth outcomes.

## Introduction

### Prenatal Exposure to Harmful Medications

The use of medication during pregnancy may be essential for the health of the mother, but some have the potential to cause harm to the fetus, which can be delayed in presentation. Prenatal exposure to harmful medications can be related to a variety of adverse outcomes, including congenital malformations, preterm birth, intrauterine growth restriction, spontaneous abortion, late fetal death, neonatal death, or developmental disabilities (behavioral, neurological, motor, intellectual, or sensory) that only become apparent in later infancy or childhood, or even later like the rare vaginal adenocarcinomas that were detected among young women who had prenatal exposure to diethylstilbestrol [1].

Unless the medicine is intended to treat pregnancy-specific conditions, at the time of initial authorization, information with respect to reproductive toxicity is usually only available from animal studies. Pregnant women are often excluded or discontinued from premarketing clinical trials in humans, so the safety of many drugs in pregnant women has not been established at the time of drug licensing [2]. Consequently, some important drugs are contraindicated or have special warnings because their safety during pregnancy has not been studied sufficiently. In addition, women who are concerned about conventional drug use in pregnancy may turn to alternative therapies, such as homeopathic or herbal medicines, which they perceive as being “natural” and somehow safer. Many of these alternative drugs are not regulated and information about safety in pregnancy is often lacking. Many pregnancies are unplanned and inadvertent exposure of the fetus to prescription or nonprescription medications may have already occurred by the time the woman realizes she is pregnant. Because organogenesis occurs early in pregnancy, first-trimester exposures are of particular interest as detrimental effects on the fetus may have already occurred before a pregnancy is confirmed [3,4]. Therefore, good postmarketing information on drug use and other possible risk factors and pregnancy outcome is needed, not only to provide information on which drugs may be unsafe, but very importantly on which drugs are probably safe.

The majority of data available on medications used during pregnancy and lifestyle factors are usually collected either from health care professionals, through direct patient questioning by an interviewer (frequently a midwife), or making use of prescription or dispensing records. In most situations, women tend to present for medical attention once it appears the pregnancy is viable, making it difficult to get accurate information about all medication exposures that occur early in pregnancy [5]. Using researchers to collect information is time consuming and expensive, and can only be performed at relatively infrequent times during the pregnancy, which may lead to lost information [6]. In addition, women may be reluctant to report accurate information about lifestyle behaviors already identified as being potentially harmful to a fetus, or which are in themselves illegal, in a face-to-face interaction. There is some evidence that using the Internet may overcome these issues related to collection of potentially sensitive information, for example, a study on sexually transmitted diseases using an anonymous Internet questionnaire successfully collected data on the number of sexual partners and cocaine use [7].

### Purpose of Pilot Study

This pilot study was designed to explore (1) whether women would be willing to volunteer for Internet-based research without any encouragement or direct involvement of their health care providers, (2) if they would provide information prospectively on exposure to medications and other factors that may affect birth outcome, and (3) if so, to assess whether this information was complete and accurate enough to be useful for pharmacovigilance.

## Methods

### Study Eligibility Requirements

To be eligible for study entry, women had to be pregnant; resident in 1 of 4 countries in the European Union (Denmark, DK; The Netherlands, NL; Poland, PL; or the United Kingdom, UK); to be proficient in the predominant language of their country of residence; to have access to the Internet or a telephone; and to be of an age to provide legal consent (16 years of age in the UK, 18 years elsewhere). Ethical review and review of data protection plans were reviewed as necessary in all countries, and the data protection plan was also reviewed by the European Medicines Agency and the European Data Protection Supervisor [8]. The study was promoted using a variety of methods including Internet announcements, email to members of pregnancy clubs, flyers placed in pharmacies, and radio and television interviews.

### Study Participants

Women were invited to respond by Internet or interactive voice response system (IVRS), using the predominant natural language in each of the 4 study countries. The IVRS system was developed in addition to Internet-based systems to facilitate participation regardless of Internet access, thus making the study available to women without access to computers, or the requisite computer skills. In NL and the UK, women could use either method for enrollment and informed consent, but in DK, informed consent by Internet was required even for subsequent participation by IVRS. In PL, the IVRS system was not offered because women were required to print, sign, and return the informed consent to the university coordinators, necessitating using the Internet system. Those who chose to participate by Internet were also offered the choice of responding to questionnaires every 2 or 4 weeks. Following study entry, women provided contact information, chose a personal identification number, and reminder method (text and/or email). Women could enroll at any time during their pregnancy, as long as it was prior to delivery, recognizing that not all women would be able to be followed throughout their entire pregnancies. Study data were collected using a study number and were maintained on a server that was separate from where contact details were stored.

Participants were recruited between October 1, 2012 (recruitment week 1) and January 31, 2014 (recruitment week 70) in DK, NL, and the UK; follow-up ended on March 28, 2014. Because of difficulties in arranging ethical approvals, the start date in PL was delayed until May 20, 2013 (recruitment week 34). Data were collected on medications used to treat illnesses including prescription and nonprescription medications, vaccines, x-rays, and various lifestyle factors, recreational drug use, and herbal products during current pregnancy or in the month preceding it; additionally, we collected basic demographics, education and ethnicity, and current and previous pregnancy history. Participants were also asked if they had used any medications that were not prescribed for them (borrowed medications) or had decided not to use medications that were prescribed. Follow-up questionnaires, provided at 2- or 4-week intervals as decided by the participant, asked about any changes to use of medications, and a final outcome questionnaire sought information about the birth outcome, including the presence of any birth defects. Participants were asked to tell us about medication use through a series of checkboxes of top 10 lists of medications that are commonly used during pregnancy specific to each country, as agreed by the study team. These lists were organized into medical conditions as a memory aid. Participants were also able to report other medication use as free texts.

In the UK, study participants were asked to provide their consent for linkage with primary care electronic health records (EHRs) in an effort to provide some validation of self-reported data, and in DK, consent for linkage with the national registries was mandatory for participation. Medications were classified according to their over-the-counter (OTC) or prescription status in DK, and applied to all other countries. Thus, medications were classified as available by prescription only (Rx only), available OTC, but may also be available by prescription in some countries depending on dose and various prescribing practices for pregnant women (OTC and Rx, or OTC or Rx), and OTC products not available by prescription (OTC only.) Those medications that were reported as being used during pregnancy and which can be obtained OTC and by prescription included acetylcysteine, acetylsalicylic acid, acyclovir, benzydamine, budesonide, fexofenedine, fluticasone, ibuprofen, lansoprazole, loperamide, pantoprazole, and paracetamol.

## Results

### Study Participants

**Figure 1:**
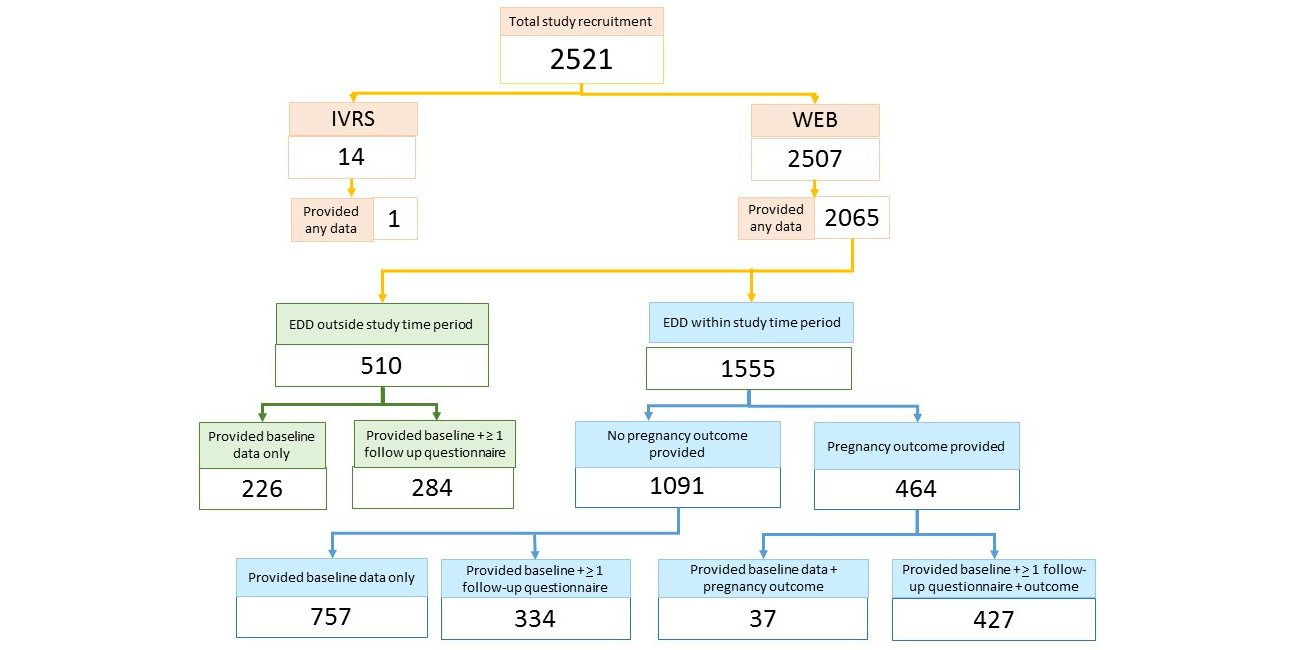
Recruitment and retention. WEB (Web, World Wide Web) is a method of accessing information over the medium of the Internet; IVRS: interactive voice response system; EDD: expected date of delivery.

Overall, 2521 women were enrolled in this study. Figure 1 shows a flowchart showing recruitment and retention through study close. There were 14 women (0.55%, 14/2521) who chose to provide information via IVRS and only 1 actually completed the baseline questionnaire, but did not provide information on birth outcome. Hence, results reported here are for those who participated via the Internet.

### Internet Results

Of 43,068 people who clicked on the website, 23,536 (54.64%, 23,536/43,068) stayed for at least 30 seconds, and 2507 (10.65%) of 23,536 women enrolled and provided informed consent (DK 770; NL 568; PL 316; the UK 853). Some women discontinued participation before completing the baseline questionnaire, leaving 2065 who completed at least baseline data (DK 639; NL 476; PL 241; the UK 709). There were 43.00% (888/2065) of those who completed the baseline questionnaire that chose to respond every 2 weeks and 56.99% (1177/2065) every 4 weeks. The age distribution of participants in comparison to the general population of each country is shown in Table 1. Most participants reported themselves to be white (95.93%, 1981/2065 in all 4 countries). The educational status of participants, shown in Table 2, reveals that participants reported being highly educated, with 38.74% (800/2065) having completed some university or postgraduate education, and a particularly high rate of postgraduate education in PL. Most women had at least one previous pregnancy that resulted in a live birth, but 42.95% (887/2065) of participants were reporting their first pregnancies (38.3%, 245/639 in DK; 47.3%, 225/476 in NL; 50.6%, 122/241 in PL; and 41.6%, 295/709 in the UK); 23.00% (475/2065) of women enrolled were in their first trimester of pregnancy, 52.34% (1081/2065) in their second trimester, and 24.65% (509/2065) in their third trimester.

**Table 1:** Age of participants and referent population in each country.

				Age in years at end of pregnancy					
				n (%)					
		Mean (SD)	n	<20	20-24	25-29	30-34	35-39	>40
**Denmark** ^a^									
	PROTECT^f^	31.5 (4.6)	639	1 (0.2)	43 (6.7)	214 (33.5)	237 (37.1)	118 (18.5)	26 (4.1)
	National	30.9 (5.1)	55,225	750 (1.35)	6192 (11.21)	17,112 (30.98)	19,319 (34.98)	9769 (17.68)	2083 (3.77)
**The Netherlands** ^b^									
	PROTECT^f^	31.3 (4.3)	476	1 (0.2)	29 (6.1)	153 (32.1)	206 (43.3)	74 (15.5)	13 (2.7)
	National 2012	30.9 (4.9)	173,085	2257 (1.30)	17,727 (10.24)	53,181 (30.72)	64,498 (37.26)	29,562 (17.07)	5860 (3.38)
**Poland** ^c^									
	PROTECT^f^	29.8 (4.2)	241	1 (0.4)	29 (12.0)	109 (45.2)	77 (32.0)	22 (9.1)	3 (1.2)
	National	29.2 (NA^a^)	370,932	14,522 (3.91)	63,158 (17.02)	131,373 (35.41)	110,192 (29.70)	43,554 (11.74)	8133 (2.19)
**United Kingdom** ^d^									
	PROTECT^f^	31.7 (5.1)	709	5 (0.7)	67 (9.4)	183 (25.8)	266 (37.5)	159 (22.4)	29 (4.1)
	England & Wales 2013	30.0 (NA^e^)	698,512	29,136 (4.17)	119,719 (17.13)	196,693 (28.15)	212,306 (30.39)	111,500 (15.96)	29,158 (4.17)

^a^The Birth Register at Statens Serum Institut; also see website [9].

^b^Stichting Perinatale Registratie Nederland: Perinatale Zorg in Nederland 2012. Utrecht: Stichting Perinatale Registratie Nederland 2013

^c^Central Statistical Office of Poland [10,11]

^d^UK Office for National Statistics, Age England & Wales 2013

^e^NA: not available

^f^PROTECT: Pharmacoepidemiological Research on Outcomes of Therapeutics by a European ConsorTium

**Table 2:** Educational levels of respondents compared with national statistics.

			Number (%) by highest education level				
		N	Legal part of school and 1st level of exams (age 16 years)	School and higher level exams	Some 3rd level education (university, etc)	University and some post graduate	Not stated
**Denmark** ^a^							
	PROTECT^e^	639	18 (2.8)	74 (11.7)	296 (46.7)	246 (38.8)	5 (0.8)
	National	56,328	8639 (15.33)	18,488 (32.88)	17,431 (30.94)	8201 (14.55)	3569 (6.33)
**The Netherlands** ^b^							
	PROTECT^e^	476	22 (4.6)	110 (23.1)	161 (33.8)	183 (38.4)	0 (0)
	National	2159	376 (17.41)	905 (41.91)	551 (25.52)	304 (14.08)	23 (1.06)
**Poland** ^c^							
	PROTECT^e^	241	4 (1.7)	21 (8.7)	56 (23.2)	158 (65.6)	2 (0.8)
	National	17.0 million	4.2 million (24.4)	8.0 million (47.1)	0.6 million (3.7)	3.3 million (19.5)	0.9 million (5.3)
**United Kingdom** ^d^							
	PROTECT^e^	709	89 (12.6)	153 (21.6)	251 (35.4)	213 (30.0)	3 (0.4)
	National	40.0 million	17.8 million (44.4)	8.7 million (21.8)	13.2 million (33)		0.3 million (0.8)

^a^Data from Statistics DK, Births 2012

^b^Data source: age: Stichting Perinatale Registratie Nederland. Perinatale Zorg in Nederland 2012. Utrecht: Stichting Perinatale Registratie Nederland, 2013; educational level: all women age 25-45, Statistics NL [12,13]

^c^The national figures for PL do not correspond completely with the educational levels used in PROTECT since level 4 for the national figures includes licentiate which is included in level 3 for PROTECT. Data source: Central Statistical Office of Poland Housing Census 2011 for women age 15 and over [10,14].

^d^The national figures for the UK are for women from 2011. The UK national figures for post graduate education not available.

^e^PROTECT: Pharmacoepidemiological Research on Outcomes of Therapeutics by a European ConsorTium

### Medication Use During Pregnancy

**Figure 2:**
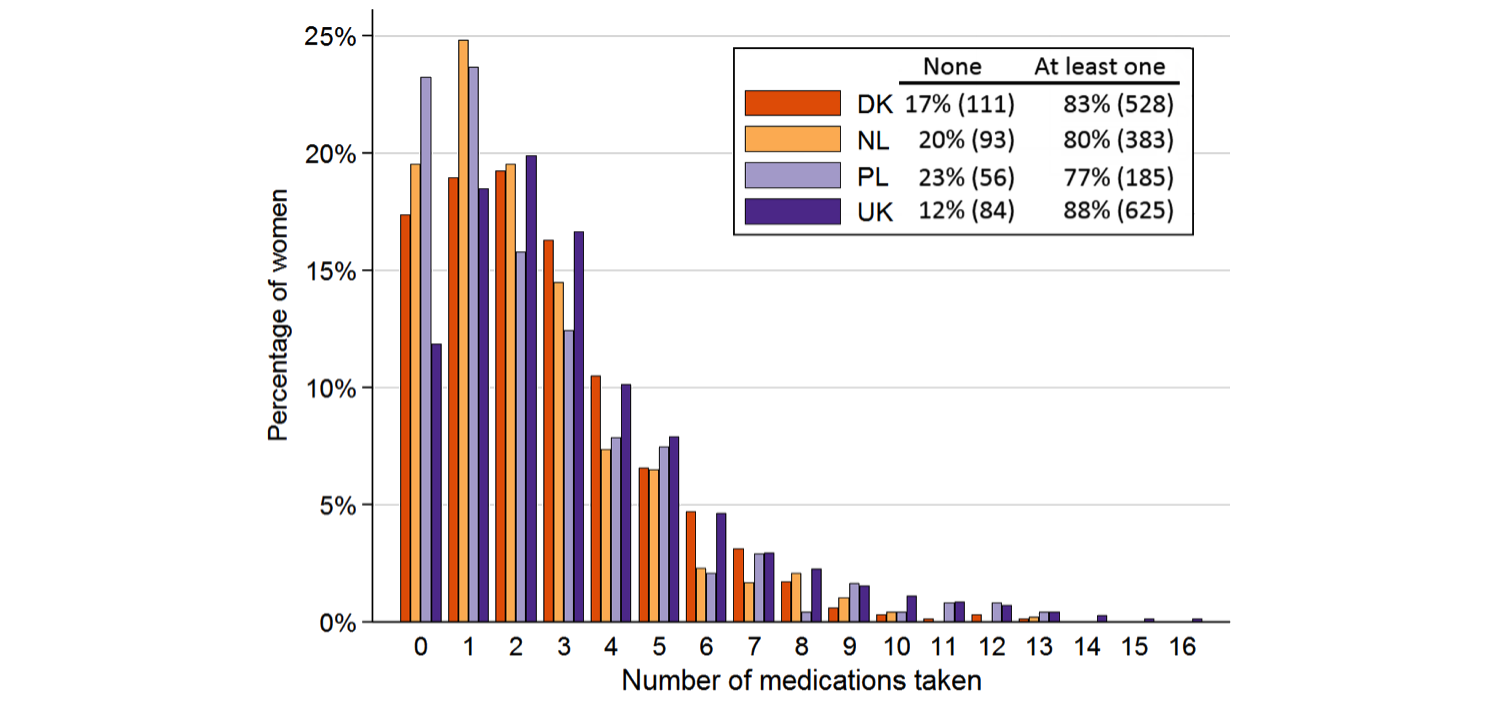
The number of different medications taken per woman by percentage in each country. Includes prescription and nonprescription medications; excludes herbals, fish oil, homeopathic, multivitamins, iron, vaccinations, and antimalarials. Denmark: DK, The Netherlands: NL, Poland: PL, United Kingdom: UK.

There were 92.7% (1915/2065) of women that reported using at least one pregnancy-related medication including fertility medications, iron tablets, multivitamins, and folic acid. Excluding those pregnancy-related medications, 83.34% (1721/2065) of women reported using 1 or more medicines (range 1-16) during pregnancy or in the month preceding it. Figure 2 shows the distribution of number of medications by woman and country. Of all reported medications, 42.46% (2333/5494) were reported as being used “as needed” and 53.82% (2957/5494) were reported as being taken daily. Recognizing that some women reported more than one reason for their medication use, the most frequent reason for all reported medication use was for nervous system disorders (71.86%, 1484/2065), followed in descending order by 62.76% (1296/2065) for alimentary tract and metabolism, 38.01% (785/2065) for respiratory issues, 30.89% (638/2065) for genitourinary system or as sex hormones, and 25.66% (530/2065) as anti-infectives for systemic use.

The top 10 most frequently used medications and medication changes during pregnancy are shown in Table 3. There were 1230 (59.56%) of 2065 women who took at least one prescription medication (Rx) and may also have used OTC medications. By contrast, 23.58% (487/2065) women reported using medications generally available OTC, but never using any medications only available by prescription. These included 7.16% (148/2065) of our respondents, who reported that they chose not to take a medication that had been prescribed for them. The two most frequently reported indications for which a woman decided not to take a medication or decreased her dose (in consultation with her caregiver) were antidepressants and anti-inflammatories. Only 1.30% (27/2065) women reported using medicines that had been prescribed for a friend or family member, but not for them. The most frequent medications that were reported as having been shared were analgesics (oxycodone and paracetamol, n=4) and drugs for acid-related problems (n=8).

**Table 3:** The 10 most frequently reported medications used during pregnancy or during the month prior to pregnancy, and changes during pregnancy (rank ordered).

		Medications used during pregnancy or month prior to pregnancy		Rank order of top 10 medications				
				Medication		Changes during pregnancy		
		n/N	%	Rx	OTC	Not taken	Increased	Decreased^a^
**Antibiotic**								
	Pivmecillinam	67/2065	3.24	5				
**Antidepressants**								
	Citalopram	34/2065	1.65			2		5
	Fluoxetine	30/2065	1.45			7		7
	Sertraline	39/2065	1.89			10^b^		8
**Antidiabetics**								
	Insulin aspart	27/2065	1.30				5	
	Metformin	48/2065	2.32	9		10^b^		
**Antifungal**								
	Clotrimazole	135/2065	6.54		5			
**Anti-inflammatories and pain**								
	Acetylsalicyclic acid	48/2065	2.32				6	
	Diclofenac	16/2065	0.77			6		10^b^
	Ibuprofen	149/2065	7.21		4	1		1^b^
	Paracetamol	1483/2065	71.82		1	4	4	1^b^
	Paracetamol combination	80/2065	3.88		9			6
	Tramadol	28/2065	1.36			5		9
**Anti-infectives**								
	Amoxicillin	165/2065	8.00	1				
	Fluconazole	64/2065	3.10	6				
**Antimigraine**								
	Sumatriptan	31/2065	1.50			3		3^b^
**Digestive disorders**								
	Metoclopramide	47/2065	2.28	10				
	Alginic acid	291/2065	14.09		2		3	
	Ispaghula (psylla seeds)	90/2065	4.36		8			
	Lactulose	101/2065	4.89		6			
	Omeprazole	80/2065	3.88		10		9	
	Ordinary salt combinations (eg, calcium carbonate)	173/2065	8.37		3			
	Ranitidine	51/2065	2.46				7	
**Ear, nose, and throat/decongestant**								
	Xylometazoline	93/2065	4.50		7			
**Reproductive**								
	Clomifene	57/2065	2.76	8				
	Ethinyl estradiol	5/2065	0.24			9		
	Hormonal contraceptives for systemic use	73/2065	3.54	4^c^		8		
	Progesterone	59/2065	2.86	7^c^				
**Respiratory**								
	Beclomethasone	30/2065	1.45				8	
	Salbutamol	96/2065	4.64	2			2	3^b^
	Terbutaline	32/2065	1.55				10	10^b^
**Thyroid**								
	Levothyroxine sodium	83/2065	4.02	3			1	


^a^Decreased, but not stopped

^b^Tied for place

^c^Probably represents use in the month before pregnancy

### Use of Anesthetics, Cosmetic Surgery, and Alternative Medicines During Pregnancy

In addition to prescription medications, 7.31% (151/2065) of women reported using an anesthetic during pregnancy, with most (n=128) having received local anesthetics. Very few women (0.44%, 9/2065) reported having undergone a cosmetic procedure during pregnancy. Relatively few women used alternative medicines and dietary supplements during pregnancy (Table 4). The most frequently cited products were herbal (7.07%, 146/2065), followed by fish oil (4.60%, 95/2065).

**Table 4:** Percentage (number) women who used alternative medications and dietary supplements during pregnancy.

Medications	n/N	All (%)
Fish oil	95/2065	4.60
Homeopathic products	24/2065	1.16
Other dietary supplements	39/2065	1.89
Herbal products	146/2065	7.07
Neither	1761/2065	85.28
Total	2065/2065	100.00

### Recreational Drugs During Pregnancy

Very few women (0.82%, 17/2065) reported using recreational drugs during pregnancy and among those recreational drug users, cannabis was by far the most widely used (65%, 11/17). The majority of women reported that they did not use any alcohol during pregnancy (76.90%, 1588/2065).

### Retention and Data Quality

Study retention was relatively low with only 464 (29.84%) of 1555 women providing information on pregnancy outcome among those with due dates (plus 1 week) occurring while the study was active (Figure 1). Those who provided some follow-up were slightly more likely to continue in the study if they were taking 5 or more medications compared to those who dropped out after baseline (45.95%, 522/1136 vs 41.4%, 385/929). Further compared with those who continued in the study, women who discontinued participation after completing the baseline questionnaire were more likely to have been smokers at baseline (6.8%, 63/929 vs 2.46%, 28/1136), and to have volunteered to provide information monthly rather than every 2 weeks (64.7%, 601/929 vs 35.3%, 328/929), and were less likely to have completed university level coursework (71.8%, 667/929 vs 78.96%, 897/1136) or to be currently taking medications (3.5%, 33/929 vs .96%, 11/1136). There were no apparent differences between age, gravidity, or alcohol use for those who dropped out quickly compared with those who provided some follow-up.

In absence of source data verification, it is not possible to confirm the accuracy and reliability of all the data provided. Nevertheless, it was possible to compare self-reported data with available information in 2 countries, because all 639 participants in DK could be linked to their EHRs using the 10-digit civil registration number, and 79.0% (674/853) of women enrolled in the UK consented to EHR linkage. In the UK, the Health Improvement Network (THIN) database of pseudonymized primary care records was compared with the patient generated records. Because THIN is only a sample of the UK population, only 18 women were successfully linked to their EHRs in THIN and had data recorded during the period when the Pharmacoepidemiological Research on Outcomes of Therapeutics by a European ConsorTium (PROTECT) study subject was pregnant; 2 of those women were still pregnant at the close of PROTECT data collection.

We compared self-reported pregnancy outcome to the EHRs in the UK for 16/18 women who had reached their due date within the study period. Of the 16 whom we were able to match, 2 (12%) had an outcome (birth) noted in both PROTECT and their EHR. There were 8 (50%) of 16 women that had a pregnancy outcome recorded on EHR only, and 3 (19%) of 16 women reported only in PROTECT. In addition, information about the pregnancy outcome was not available in either PROTECT or the EHR for 3 of the 16 women (19%). It is possible that the THIN EHR may have been updated with information on their pregnancy outcomes after the data were provided for this study.

When data were compared from PROTECT with similar information for the women in the Danish Pharmacy register and from THIN in the UK (Table 5), some underreporting of medications was evident for women in PROTECT for chronic conditions, short-term conditions, and pregnancy-related medications. A small number of women also reported not taking prescribed medications, which may explain some differences between self-reports and database reports.

**Table 5:** Compatibility and discrepancy between PROTECT self-reported prescription use and electronic health care data in DK and the UK based on the whole follow-up period, N_PROTECT_/N_DATABASE_ (%).

	DK n=639		UK n=18
	All, n (%)	Excluding dispensed, but not taken^a^, n (%)	All^b^, n (%)
Drugs for chronic conditions	145/174 (83.3)	145/170 (85.3)	3/6 (50)
Drugs for occasional or short-term use	153/283 (54.1)	153/276 (55.4)	4/12 (33)
Pregnancy-related medications	78/118 (66.1)	78/114 (68.4)	6/10 (60)

^a^Adjusted for self-reported decision not to take a prescribed medication.

^b^None of these respondents in the UK reported a decision not to take a prescribed medication.

## Discussion

### Principal Findings

There is incomplete information about the efficacy and safety of treatments used by the broad array of patients seen in everyday clinical practice [15]. Could direct-to-patient research help fill that gap for pregnant women with information that could be used to investigate possible risk factors for negative birth outcomes in order to guide expectant mothers and their clinicians to make healthy pregnancy choices? Could direct-to-patient data also be used to understand treatment benefits and risks to inform decisions about personalized medicine [16]? This study shows that some pregnant women will volunteer to participate in pharmacovigilance-type studies and will report medications used in pregnancy, including both prescription and nonprescription medications, as well as other life style factors, like alcohol and drug use. A comparison of our respondents with demographics from each of their countries shows that our volunteers were broadly representative of the population density in each country, as well as age and parity, although they were more educated than their peers, and in some countries, were not as ethnically diverse as the underlying population (eg, 94.5%, 670/709 white in this study vs 86%, 48.2 million/56.1 million in the UK) [17]. To the extent that biologic responses to medication are not heavily dependent on education and ethnicity, these data would support the use of direct-to-patient research, as it may have benefits for understanding both safety and effectiveness. Further, it appears that interactive voice response is not a popular method for consumer responses. In this study, the IVRS was only chosen by 14 patients and of those, only 1 provided any usable data.

### Recruitment and Retention

Loss to follow-up was high in this study, which without substantial improvement would lessen the value of direct-to-patient research for pharmacovigilance. This study, however, employed very minimal patient reminders, only unvarying reminder emails and/or text messages a few days before, and once after, a follow-up form was expected. No patient incentives were used and the Web study portal only provided study-related information and no other information to attract or maintain the interest of study participants. Using only these straight forward methods for retention, only 55.01% (1136/2065) of the subjects who enrolled in this study provided any follow-up, which may reflect, in part, the difficulty of filling out a detailed questionnaire and perhaps the unwanted focus on lifestyle factors that may negatively affect birth outcome. Only 29.83% (464/1555) of the women provided information about birth outcome. Among those who provided some follow-up, the loss-to-follow-up rates were more than doubled among women who enrolled in the second and third trimesters when compared to those who enrolled in the first trimester.

To further explore reasons that would affect recruitment and retention, we convened a small focus group of 45 first-time mothers in their first pregnancy from the DK, NL, PL, and the UK to assess their willingness and interest in participating in a study like PROTECT. Although most women reported that they would participate in a study like this purely for altruistic reasons, one of the most frequent comments was that some form of modest compensation would enhance the appeal of participation.

### Prescription Reporting

Getting detailed, interpretable reports about medication use was challenging. We asked about medication use according to the indication for which it was being used, did not ask about dosage or route of administration, and did not distinguish between prescription and nonprescription medication because that varied from country to country and could change at any time during the study. Instead, we used a list of nonprescription medication for DK (the only country that was able to provide such a list) and applied that assumption to the other 3 countries, recognizing that in some countries, pregnant women receive prescriptions for medications that are available OTC in order to have those medications paid for by their insurance. We provided machine-prompts on the questionnaires for the top 10 medications in each country for each indication to guide data entry for medications, which was challenging to compile in 4 countries as proprietary names of the same products varied by country and by whether a prescription was required for a particular medication and dose. Nearly 25% (3136/12,699) of the medications used were reported as free text, rather than using the drop-down list available on the questionnaire for this purpose, which required substantial manual review and recoding. More consumer-friendly computerized methods of acquiring data about medication use would be helpful for future research.

### Strengths of Direct-to-Patient Research

Despite the challenges we encountered in distinguishing medications that were obtained by prescription and those that were not, the extensive reporting of medication use here is a strong advantage for understanding the causal relations of drugs and adverse events. About 4 of 5 women (80%) reported using at least one medication during or immediately before pregnancy, a figure that is in line with some other estimates [18-22] and higher than others (eg, 50%) [23]. Our estimates may be higher than some other studies because our recruitment materials and informed consent documents described our interest in understanding medication use during pregnancy. Nonetheless, the information that our respondents provided corresponds fairly well with other reports. For example, a recent study of medications used by Medicaid recipients during pregnancy showed the most commonly dispensed medications during pregnancy were antibiotics and anti-infectives (nitrofurantoin, 21.6%; metronidazole, 19.4%; amoxicillin, 18.0%; azithromycin, 16.9%) and an antihistamine (promethazine, 13.5%) [23], whereas another US study showed the most commonly used prescription medication components during the first trimester of pregnancy were progestins from oral contraceptives, amoxicillin, progesterone, albuterol, promethazine, and estrogenic compounds [18]. In our study, the most common prescription medications were anti-infectives (amoxicillin) and antibiotics (pivmecillinam), as well as respiratory medication (salbutamol), thyroid medication (levothyroxine sodium), and hormonal medication (contraceptives). Interestingly, we noted that respondents might not always understand the indication for which they are taking prescribed medications. For example, in our validation study in the UK, physicians reported prescribing no antidiabetic medications for the 18 women whose records were matched with PROTECT, yet 1 woman self-reported taking an antidiabetic medication.

Our estimate that 40.73% (841/2065) of pregnant women used nonprescription medicine is not much different from the limited published literature [19], even though the reference estimate may not include the identical nonprescription medications that were used in this study. In the US study of medications used during the first trimester of pregnancy, the most commonly used OTC medications included anti-inflammatories and pain medications (acetaminophen and ibuprofen) and medication for digestive disorders (docusate) [18], as did our study (paracetemol and ibuprofen; alginic acid and ordinary salt combinations like calcium carbonate). Of note, the methodology used here provided rich information about intermittent use of medication as well as nonprescription and complementary medication use, the details of which are rarely available from other sources. This pilot study gives some indication that women are willing to report their use of herbal medications, although the estimates from these 4 study countries (7.07%, 146/2065 of our respondents self-identified as using herbals during a pregnancy) are lower than the 6% and 11% reported in 2 US studies [20,21], and the 55% reported in Italy in 2009 [22].

### Conclusions

Taken as a whole, it appears that direct to consumer (or patient) is a useful method for learning about use of prescription and nonprescription medication use, including medications that may be administered in hospitals, emergency rooms, or as outpatients, and in some cases, these patient-reported data are more complete than reliance on existing data like prescription registers and EHR [23]. Further, using the Internet to collect data directly from patients/consumers makes it easier to collect data regularly during a study. This steady data collection may reduce recall bias, which is particularly useful when investigating potential effects of medication use during pregnancy on birth outcomes, since such effects are largely dependent on gestational age at the time of exposure. In addition, because women could enter the study as soon as they became pregnant, it provides valuable information on exposure during the early weeks of pregnancy, which might not be available, or complete, using more traditional interview methods.

Could patient-reported information be a complete substitute for other types of pharmacovigilance? Although there appears to be some underreporting about medication use and perhaps herbals, self-reporting of prenatal exposures provides a more complete picture of factors which may contribute to an adverse outcome than does reliance purely on existing traditional methods of collecting data. The value of having information on medication use as well as other exposures that may not be readily available is appealing, for example, anesthesia, travel vaccinations, as well as other behaviors that patients may be reluctant to report directly to their physicians, such as use of recreational products like cocaine and marijuana. Such a rich data source would enhance our ability to evaluate individual teratogens as well as to look at various exposures, which may be risky when used in combination during pregnancy. Nonetheless, there are shortcomings to sole reliance on patient-reported data that make it difficult to use in the absence of supplementary data, including (1) accurate and complete product reporting of molecular entity, dose, and manufacturer; and (2) clinician-reported assessment of birth outcomes. Looking toward the future, perhaps the most effective method to assemble meaningful information about potential teratogens would be to combine patient-reported data with information on prescription and/or clinical validation from physicians or health care registers for major events and exposures of special interest.

## Abbreviations

DK: Denmark
EHRs: electronic health records
IVRS: interactive voice response system
NL: the Netherlands
OTC: over-the-counter
PL: Poland
PROTECT: Pharmacoepidemiological Research on Outcomes of Therapeutics by a European ConsorTium
UK: United Kingdom
